# Antecedents of Viewers’ Live Streaming Watching: A Perspective of Social Presence Theory

**DOI:** 10.3389/fpsyg.2022.839629

**Published:** 2022-03-31

**Authors:** Jiada Chen, Junyun Liao

**Affiliations:** Research Institute on Brand Innovation and Development of Guangzhou, School of Management, Jinan University, Guangzhou, China

**Keywords:** sense of community, emotional support, interactivity, streamer attractiveness, social presence, live streaming

## Abstract

Live streaming commerce as a popular marketing method has attracted wide attention, but little is known about why consumers continue to watch live streaming. To fill this research gap, this study draws on social presence theory to examine the impact of sense of community, emotional support, and interactivity on viewers’ social presence, which, in turn, influences their live streaming watching. Furthermore, the moderating role of streamer attractiveness is also investigated. The authors collected survey data from 386 live streaming viewers and used the structural equation model to test the research model. The results reveal that sense of community, interactivity, and emotional support positively affects viewers’ social presence, leading to viewers’ watching live streaming. Furthermore, streamer attractiveness plays a significant moderating role between social presence and live streaming watching. This study provides a unified theoretical framework to explain the intention to watch live streaming based on social presence theory.

## Introduction

Live streaming has attracted much attention as an emerging form of online media because of its unique sense of immediacy and interactivity (Xue et al., 2020). It facilitates real-time interaction between streamers and users and thus is increasingly adapted into social commerce for production introduction and promotion, resulting in so-called live streaming commerce (Niedermeier et al., 2016; Shanmugam et al., 2016; Florenthal, 2019). The apparent advantage of live streaming is that streamers can interact and communicate with viewer’s through virtual face-to-face communication *via* live streaming (Huang and Benyoucef, 2015). In real-time interaction, the viewer’s product experience and sense of presence are significantly enhanced, providing the prerequisite for continuous watching and purchasing decisions.

In recent years, the widespread use of live streaming has attracted academic research. Current research focuses on viewer’s motivation to watch live streaming and the purchase decision process (Bründl and Hess, 2016; Hamari and Sjöblom, 2017; Hu et al., 2017; Chen and Lin, 2018) and examine the impact of live streaming on viewer’s purchase intention (Sun et al., 2019; Zhou F. et al., 2019; Zhang et al., 2020). Different from traditional marketing media, social presence is the most significant advantage of live streaming (Xue et al., 2020), so the role of social presence has been widely mentioned in the research on live streaming (Hu et al., 2017; Xie et al., 2019; Liu et al., 2020). Nevertheless, we discover that there are still limitations in the research on social presence in live streaming. First, regarding viewers watching live streaming, current research mainly considers viewers’ motivations, such as hedonism (Hilvert-Bruce et al., 2018; Yu et al., 2018; Xu et al., 2019; Ma, 2021), utilitarianism (Ma, 2021) and social status needs (Hilvert-Bruce et al., 2018; Wongkitrungrueng and Assarut, 2018; Hou et al., 2019; Ma, 2021), or system functions, such as interface design (Sun et al., 2019; Kim et al., 2020). However, live streaming is a process of communication. How live streaming affects viewers’ experience and, thus, their behavior is rarely explored in this communication process. Second, studies on the antecedents of social presence in live streaming are primarily considered in terms of system functionality (Sun et al., 2019; Kim et al., 2020) and gift visualization (Yu et al., 2018; Zhou J. et al., 2019), but lack how the connections and interactions between viewers affect social presence. Social presence is an experience and feeling brought by live streaming. It is often generated by communication and interaction between viewers and streamers or other viewers, such as emotion, live community atmosphere, and interaction (Ang et al., 2018; Chen and Lin, 2018; Li, 2019). Revealing the formation and outcome of social presence in live streaming commerce is of theoretical and managerial importance.

Social presence theory explains the salience of human perception of others’ communication in media and interpersonal interactions (Biocca et al., 2003; Lowry et al., 2009). Unlike physical presence, social presence emphasizes communication and interaction in online media (Xu et al., 2021). However, the perceived coexistence of others is a prerequisite for the emergence of social presence (Lee, 2004). Further research on social presence theory has found that social presence is conceptualized as three dimensions: coexistence, psychological connection, and behavioral involvement (Garrison et al., 1999; Biocca et al., 2003). Coexistence refers to perceiving the presence of others and responding to them (Shen and Khalifa, 2009). In online communities, the specific application is to perceive the presence of the community and its interactions (Garrison et al., 1999; Whiteside, 2015). This is because users tend to coexist through perceived communities or groups in practice. In addition, psychological connections are mainly made through emotional communication (Biocca et al., 2003). In contrast, behavioral engagement mainly refers to communication and interaction between users. In online communities, emotional expression, open communication, and group cohesion are seen as key to the social presence of the community (Garrison et al., 1999). Moreover, emotional connectedness, community cohesion, and interactivity have also been confirmed for their role in enhancing social presence in online communities (Whiteside, 2015). In other words, the formation of social presence involves emotion, community cohesion, and interactivity. Therefore, in the context of live streaming, we specifically outline sense of communitys (users perceive the existence of a live streaming community and its cohesiveness), emotional support (emotional expression and communication), and interactivity (sending pop-ups and participating in activities) (Mamonov et al., 2016; Wohn et al., 2018; Al-Emadi and Ben Yahia, 2020).

Therefore, we examine how these three variables affect social presence in live streaming. In addition, there is another critical factor in live streaming: the streamer. The streamer engages the viewers by organizing and conveying the content. Although we explore the formation of social presence from the viewer experience perspective, it is inevitably influenced by the streamer. Attractiveness embodies streamers’ charm and comprehensive ability (Wiedmann and von Mettenheim, 2020). Therefore, streamer attractiveness is selected as the moderating variable. We use data gathered through an online survey (*N* = 386) to test our hypotheses. Sense of community, interactivity and emotional support positively influence viewers’ social presence, and in turn, social presence influences watching intention. The results of the moderation test show that streamer attractiveness positively moderated the relationship between social presence and watching intention.

This study has the following three theoretical contributions. First, we contribute to the literature explaining intention to watch live streaming by focusing on viewer social presence in the live streaming. Second, we explain the antecedents of social presence based on viewers’ experience and combined with social presence theory. Third, we verify the moderating role of streamer attractiveness in social presence and watch intention, explaining the impact of streamer attractiveness on viewers’ perception. Finally, this study has practical value by investigating viewers’ live streaming from a social presence theory perspective and giving new insights for live streaming commerce.

## Literature Review

### Live Streaming

Live streaming is online and real-time dissemination on the Internet where video information can be captured, published, and viewed simultaneously (Zhou F. et al., 2019). It has the advantages of facilitating viewer interaction, providing viewer engagement, and meeting cognitive viewer needs (Yu et al., 2018; Shen, 2021). In addition, the social presence and synchrony in live streaming enhance the viewers’ experience more than pre-recorded video (Ang et al., 2018).

Live streaming has become a novel way and method of e-commerce. Live e-commerce has social and e-commerce attributes (Chen and Lin, 2018). Live streaming becomes the front end of merchandise shopping and socially connects a wide range of consumers. Companies using live streaming can achieve marketing purposes and increase the potential of communicating with existing and prospective customers (Wang et al., 2016). Adopting a live selling strategy was more effective by 27.9% for sellers of experiential goods than for sellers of tangible goods (Chen et al., 2019). Moreover, live streaming content offers viewers entertainment and social interaction value. For instance, viewers can give “likes” and virtual gifts to streamers (Bründl and Hess, 2016). As a result, more viewers are drawn to interesting live content and continue to enjoy live streaming (Chen and Lin, 2018; Shen, 2021). In addition, viewers immersed in live streaming result in paid behavior and continuous positive behavior (Yu et al., 2018; Gong et al., 2020).

Scholars have studied the motivation of viewers in watching live streaming from different perspectives, and various theories concentrate on motivational drivers such as emotional (Xu et al., 2019; Lim et al., 2020) cognitive (Hilvert-Bruce et al., 2018; Xu et al., 2019), utilitarian (Ma, 2021), hedonic motivations (Hilvert-Bruce et al., 2018; Yu et al., 2018; Xu et al., 2019; Ma, 2021), socialization motivation (Hilvert-Bruce et al., 2018; Wongkitrungrueng and Assarut, 2018; Hou et al., 2019; Ma, 2021), social cognitive (Lim et al., 2020), social identification (Hu et al., 2017; Zhou F. et al., 2019), and the fit between streamers and viewers (Park and Lin, 2020). Scholars generally agree that viewers are influenced by social identification in live communities (Hu et al., 2017). Under the influence of the community, viewers are interested in the live streaming community and streamers and satisfy their curiosity through continuous watching (Wongkitrungrueng and Assarut, 2018; Zhou F. et al., 2019; Park and Lin, 2020; Shen, 2021). People also satisfy their emotional needs and assert their social status in the community, which motivate their continual live streaming watching (Chen and Lin, 2018; Wang and Wu, 2019; Zhang et al., 2020).

### Social Presence

Social presence refers to the extent to which viewers perceive that they are connected and interacting with others as independent and genuine individuals in the use of media products (Albertson, 1980). Social presence indicates the interaction degree and authenticity of the online environment (Ogara, 2014). It is often used to explain individuals’ cognitive and emotional behaviors (Han, 2016; Kim and Song, 2016), especially in social commerce platforms. Current research on social presence in the marketing field focuses on consumer-brand relationships, behavioral motivations (Kim et al., 2020; Nadeem, 2020; Obeidat et al., 2020), and online community building (Nadeem, 2020). Social presence in social media enhances viewers’ enjoyment and willingness to sustain behavior by enhancing commitment and trust in online communities (Choi, 2016; Nadeem, 2020). Moreover, social presence also provides viewers with hedonic and social benefits, leading to positive attitudes toward online communities such as belonging (Gao, 2017; Weidlich and Bastiaens, 2019; Ma, 2021).

There is research on the motivation of live streaming intention from social presence. Li et al. (2018) reveal that social presence increases the viewers’ willingness to consume virtual gifts. Su et al. (2020) find that social presence enhances the online visibility of virtual gifts as its primary mechanism. In addition, social presence can also ease the antagonistic relationships in the live streaming community (Lin, 2021). Although these studies provide insights into the effect of social presence on gift behaviors, research on the antecedents of social presence and its effect on continual live streaming watching is relatively lacking. Moreover, we notice that scholars mainly emphasized the impact of the visual scene (Liu et al., 2020) and website design (Sun et al., 2019; Kim et al., 2020) on social presence, while the role of streamers is rarely mentioned.

## Hypotheses Development

### Research Framework

**FIGURE 1:**
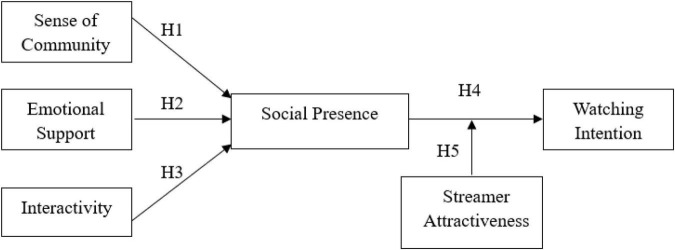
Conceptual model.

Based on social presence theory, we take emotional support, sense of community, and interactivity as the variables that affect social presence studies and then explore viewers’ watching intention (see Figure 1). In addition, we introduce streamer attractiveness as a moderating variable of social presence and watching intention to reveal how streamer attractiveness affects viewers’ watching intention.

### Sense of Community and Social Presence

Sense of community is a person’s perception of being a part of a community (Koh and Kim, 2004). Numerous studies in sociology have established that a sense of community is a critical asset in shortening the distance between people and strengthening ties within groups (Vieno et al., 2013). Besides, sense of community is conducive to forming a collective identity (Malinen, 2015). Moreover, this connection with the community can provide social benefits such as social integration and resource sharing (Bi, 2019).

Live streaming communities are different from general communities in that they pursue more participation, interaction and communication among members (Wang and Li, 2020). In addition, the most crucial feature of live communities is their inclusiveness and openness. Viewers can leave or enter at any time. Those who remain tend to have a strong emotional connection and sense of belonging to the live streaming community (Chen and Lin, 2018; Wang and Wu, 2019; Zhang et al., 2020).

Live streaming viewers meet their own needs through community participation, interaction, and socialization (Hamari and Sjöblom, 2017) and increase community engagement (Mamonov et al., 2016). Sharing, recommendations, and interaction help develop connections among participants and strengthen the sense of belonging to the live streaming community (Bi, 2019). When viewers have a high sense of community, they tend to pay extra attention to what is happening and information in the community and see it as relevant. As a result, the viewer’s perceive the live community as real-life (Xie et al., 2019). In summary, when viewers’ sense of community is higher, viewers’ social presence also increases. Thus we hypothesize:

**H1** Sense of community positively influences social presence.

### Emotional Support and Social Presence

Emotional support is about allowing people to listen, care, empathize, provide reassurance, and make people feel valued, loved, and cared for Helgeson (2003). Emotional support is a common type of social support closely related to emotional needs (Shensa et al., 2020). It effectively reduces perceived risk to users and promotes emotional solidarity (Joo et al., 2021). Emotional support may alleviate the prevalence of depression and improve the quality of life (Shensa et al., 2020). Viewers who lack emotional support in the real world tend to find emotional support in social media, which increases the willingness to sustain social media engagement (Brailovskaia, 2019). With the advent of e-commerce, the main objective of streamers and merchants has become how to increase the emotional support and recognition received by viewers. Emotional support influences viewers’ hedonism and improves their social presence (Xu et al., 2019), motivating them to watch live streaming and become loyal fans of that streamer, generating consumption behavior.

In social media, viewers can satisfy their needs through emotional support and become attached to social media (Li, 2019; Lin et al., 2021). Emotional motivation is closely related to the amount spent watching live streaming. Viewers are more likely to engage in positive, persistent behaviors when emotionally supported live streaming (Hamari and Sjöblom, 2017). Furthermore, emotional interaction and support can make viewers feel their needs are met and mentally resonate (Yuksel and Labrecque, 2016), reducing social distance and increasing the sense of authenticity and experience (Xu et al., 2019). Therefore, when the emotional support received by the viewers is higher, the psychological connection of the viewers is enhanced, which in turn increases the social presence. Thus we hypothesize:

**H2** Emotional support positively affects social presence.

### Interactivity and Social Presence

Interactivity refers to interaction in communication (Bonner, 2010; Florenthal, 2019). In social media, two dimensions are divided according to the intensity and richness of interaction: responsiveness and personalization (Kang et al., 2021). Responsiveness represents how quickly an individual responds to and processes information, whereas personalization represents how viewers interact according to their preferences. The advent of live streaming has led to an unprecedented level of social interaction for viewers (Hamari and Sjöblom, 2017). The critical interactive behaviors in live streaming include liking, recommending content, giving virtual gifts, and sending pop-ups (Yu et al., 2018; Wang and Li, 2020), personalized, responsive, and entertaining (Xue et al., 2020).

Interaction between viewers in live streaming can satisfy interpersonal needs, reduce loneliness and psychological distance, reduce perceived risk, enhance viewers’ perception of usefulness and the self-connection with the streamer (Kim and Kim, 2019; Corrêa et al., 2020), and promote a cheerful willingness to continue watching and purchasing (Yu et al., 2018; Zhou J. et al., 2019; Xue et al., 2020; Ma, 2021). Moreover, with extensive interactivity, the viewers are connected to the live streaming community more closely. Thus, we infer that interactivity effectively increases viewers’ social presence:

**H3** Interactivity positively affects social presence.

### Social Presence and Watching Intention

Social Presence may increase live streaming watching in several ways. First, social presence enhances viewers’ enjoyment (Choi, 2016; Liu et al., 2020) and positively affects viewers’ sense of belonging (Gao, 2017). Second, increased social proximity can increase viewers’ trust in online merchants (Chen et al., 2020) and shorten the psychological and social distance (Zhou F. et al., 2019). Third, social presence can positively influence online viewers’ sociability and emotions (Weidlich and Bastiaens, 2019) to attract further engagement from online members (Han, 2016; Kim and Song, 2016). Finally, social presence can increase viewers’ perception of trust and generate sustained behavior by influencing commitment and loyalty in online communities (Nadeem, 2020). Thus we hypothesize:

**H4** Social presence positively affects watching intention.

### The Moderating Effect of Streamer Attractiveness

Guo et al. (2022) define streamer attractiveness as the viewer’s perception of the streamers’ appearance, expertise and communication style. A sense of humor, friendly communication, unique skills, and engaging content format can all be unique attractiveness for streamers (Woodcock and Johnson, 2019; Al-Emadi and Ben Yahia, 2020). A streamer’s unique appeal can attract viewers to tune in and create a connection and willingness to consume (Ledbetter and Meisner, 2021). An attractive streamer increases interactivity in social media and enhances viewers’ perceived source credibility, generating continuous positive behaviors (Chahal and Rani, 2017; Wiedmann and von Mettenheim, 2020).

People spontaneously identify with streamers with particular appeal and see them as role models (Wang and Scheinbaum, 2018; Hou et al., 2019; Song and Kim, 2020). Attractive streamers can divert viewers’ stress and gain positive emotions (Hung, 2014; Yuan and Lou, 2020). Streamers attract different viewers groups through their language style, interaction skills, ability to regulate the atmosphere, and values (Xu et al., 2020). Streamers create a sense of connection and intimacy with viewers through appealing characteristics such as their sense of humor, friendly interaction, and unique content. And this satisfies viewers’ cognitive and emotional needs and attract them to continue watching and paying for live streaming (Zhao et al., 2018; Woodcock and Johnson, 2019; Park and Lin, 2020). Therefore, streamer attractiveness has a moderating effect on viewers’ watching intention. Thus we hypothesize:

**H5** Streamer attractiveness has a positive moderating effect on the relationship between social presence and watching intention.

## Methodology

### Questionnaire Design and Measurement

We adopted a survey approach to test our framework and established measurements of constructs in previous studies used in the survey. We adapted the measurement items into live streaming context to generate an appropriate questionnaire. The measure of emotional support was primarily referenced to Li (2019). The measurement of sense of community is mainly referenced to Sjöblom and Hamari (2017). The measurement of interactivity is referred mainly to Chen and Lin (2018). The measurement of social presence is mainly referred to Gefen and Straub (2004) and Sun et al. (2019). The measurement of streamer attractiveness is primarily referred to Park and Lin (2020). Finally, the measurement of watching intention is mainly referred to Chen and Lin (2018). The Likert five-point scale was used for all measurement items. Given that the questionnaire data were collected in China, the translation method we follow is Ares (2018) to maintain the validity of the original items. To ensure that respondents had some live streaming experience, only respondents who had watched live streaming four times within 1 month before the survey was qualified for participation. In addition, we added gender, age, monthly income, education level, and frequency of live viewing as control variables.

### Data Collection and Sample Description

We use Credamo, a professional online questionnaire platform, to distribute the online questionnaire. As mentioned before, we tested our hypothesis by sending questionnaires to the viewers’ who have watched the live streaming before. The collection of questionnaire data relies on Credamo’s sampling service. We collected 413 questionnaires and some were removed because of obvious logic inconsistency, remaining 386 valid ones with a valid response rate of 93.46%. The sample size was approximately 25 times the number of constructs. Respondents were mainly from two famous live-streaming platforms in China, Tmall, and Sina. We conducted independent sample *t*-tests and found no significant differences between them.

Table 1 shows the demographic information characteristics of the valid samples. Overall, the information on the demographic characteristics of the respondents matched the characteristics of the viewers watching live streaming.

**TABLE 1:** Demographics of respondents (*N* = 386).

Demographic variables	Frequency	Percentage
**Gender**		
Male	197	51.04
Female	189	48.96
**Age (Years)**		
<18 years old	13	3.37
18–23 years old	159	41.19
23–35 years old	183	47.41
35–50 years old	31	8.03
**Income (monthly/yuan)**		
Under 3,000	41	10.62
3,000–5,000	157	40.67
5,000–8,000	143	37.05
8,000 or more	45	11.66
**Education**		
High school and below	83	21.50
College and bachelor’s degree	258	66.84
Master’s degree or above	45	11.66

### Data Quality Inspection

As per Anderson and Gerbing (1988), confirmatory factor analysis (CFA) effectively tests the validity of the data and validates the model. Before this, we tested the data for normality. The skewness of most variables was below 3, while the kurtosis estimate was 5. This indicates that the data have good normality and are less affected by heteroscedasticity (Nevitt and Hancock, 2000). And then, CFA was run. Table 2 shows the test results. CR was above 0.837. Moreover, the AVE of any variable in the data was more significant than 0.631, which was in line with the standard of previous academic research (Anderson and Gerbing, 1988) and passed the discriminant validity test. Therefore, the model has good convergent validity.

**TABLE 2:** Results of confirmatory factor analysis.

Conception	Title item	Factor loading
Sense of community Cronbach’s α = 0.846 CR = 0.900 AVE = 0.687	Being a part of the live streaming room is essential to me.	0.834
	I spend a lot of time with the members of the live streaming room and enjoy being with them.	0.821
	I want to stay involved in the live streaming room for a long time.	0.828
	The members of the live streaming room share a common interest and share important things.	0.831
Interactivity Cronbach’s α = 0.882 CR = 0.906 AVE = 0.762	I will send pop-ups and give feedback.	0.872
	I will respond to the streamer’s request and give feedback.	0.884
	I will like, give gifts, and share my feelings.	0.863
Emotional support Cronbach’s α = 0.819 CR = 0.837 AVE = 0.631	Some of the viewers in that live streaming room supported me when I was in trouble.	0.797
	Some viewers in this live streaming room comforted and encouraged me when I was in trouble.	0.804
	When I was in trouble, some viewers in the live streaming room expressed their concern for me.	0.781
Streamer attractiveness	The streamer gave me a good impression.	0.842
Cronbach’s α = 0.838	The streamer is very charming.	0.837
CR = 0.880	The streamer captivated me.	0.848
AVE = 0.710		
Social presence Cronbach’s α = 0.867 CR = 0.878 AVE = 0.705	I can feel a sense of contact with viewers in the live streaming.	0.857
	I can feel a sense of socialization in the live streaming.	0.835
	I can feel a sense of human warmth in the live streaming.	0.827
Watching intention Cronbach’s α = 0.889 CR = 0.889 AVE = 0.727	I intend to continue watching live in the future.	0.852
	I plan to continue to watch the live stream regularly.	0.848
	I will always try to continue watching live streaming.	0.837

Finally, reliability and validity also were tested. In this study, the Cronbach’s α coefficient was used to assess the internal consistency of the data, and the mean refined variance method was used to evaluate the convergent validity and discriminant validity. The data test results showed that the Cronbach’s α values of all the constructs were above 0.819. Furthermore, according to the study results, the reliability was significant when the Cronbach’s α coefficient exceeded 0.8 (Jones et al., 1968), which indicated that the internal consistency of each construct was high. Furthermore, the data test results show that the square root value of the AVE was more significant than the correlation coefficient between this variable and other variables, indicating that each variable has good discriminative validity. In conclusion, the survey data has high reliability and validity and thus can be analyzed for hypothesized effects.

### Common Method Bias and Multicollinearity Test

Common method bias is a common problem in survey research. Therefore, this study followed Malhotra et al. (2006) and adopted anonymous responses to avoid this phenomenon. In addition, to determine the presence of common method bias, this study subjected all items to unrotated principal component factor analysis. Podsakoff et al. (2003) suggested that the probability of common method bias will be low if a single factor explains only < 50% of the variance. However, the results showed that the highest variance explained by a single factor was 39.15% (<50%), which could not explain most of the variance in the study (Malhotra et al., 2006). Therefore, there was no serious common method bias in this study.

We conducted a multicollinearity test based on variance inflation factor (VIF) (Cenfetelli and Bassellier, 2009; Hair et al., 2012). The results (as shown in Table 3) reveal that most VIFs are between 1.624 and 2.814, suggesting that multicollinearity is not a problem in this study (Grewal et al., 2004). Table 4 present the correlation matrix of variables.

**TABLE 3:** Result of multicollinearity test (VIF).

Conception	Items	VIF
Sense of community	SC1	1.733
	SC2	1.691
	SC3	1.724
	SC4	1.742
Interactivity	I1	2.066
	I2	2.134
	I3	1.976
Emotional support	ES1	1.624
	ES2	1.716
	ES3	1.682
Social presence	SP1	2.732
	SP2	2.814
	SP3	2.726

**TABLE 4:** Correlation matrix of latent variables.

Variables	1	2	3	4	5	6
1. Emotional support	1					
2. Sense of community	0.482**	1				
3. Interactivity	0.536**	0.327*	1			
4. Social presence	0.337**	0.347*	0.587**	1		
5. Watching intention	0.341**	0.422**	0.563**	0.551**	1	
6. Streamer attractiveness	0.379*	0.359**	0.445**	0.532**	0.436**	1
Average value	3.818	3.916	4.107	4.192	4.127	3.927
Standard deviation	0.581	0.613	0.649	0.727	0.736	0.723

*p < 0.05, **p < 0.01.

### Hypothesis Test

We conduct structural equation modeling to verify the hypotheses. The analysis data showed that the fit indicators of the study model were: x^2^/df = 2.128, RMSEA = 0.0543, NFI = 0.904, CFI = 0.913, IFI = 0.908, TLI = 0.894. The RMSEA was below the critical value of 0.06. And the model fit was acceptable (McNeish et al., 2018). The sample size in this study was 386, and the x^2^/df ratio was below 3, thus fitting a good model correlation. The CFI was 0.913, a strong fit indicator. The IFI was 0.908, another strong fit indicator. In summary, this study’s model fit and fitness are high enough to perform path coefficient analysis. Table 5 shows the results.

**TABLE 5:** Hypothesis test results.

Hypothesis	Path model	Standard path coefficient	Standard deviation	*P*-value	Hypothesis test
H1	Sense of community → Social presence	0.423	0.063	***	Support
H2	Emotional support → Social presence	0.384	0.072	***	Support
H3	Interactivity → Social presence	0.516	0.094	***	Support
H4	Social presence → Watching intention	0.634	0.085	***	Support

***p < 0.001.

**FIGURE 2:**
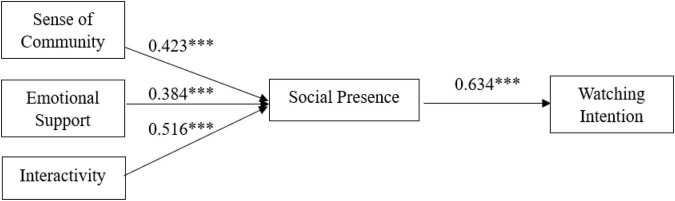
Path coefficient test results. ^***^p < 0.001.

According to the results o, the standardized path coefficients between the elements of each variable were more significant than 0.384, which passed the significance test (Wright, 1960). Therefore, all hypothesized relationships were valid. Sense of community (β = 0.423, *p* < 0.001), emotional support (β = 0.384, *p* < 0.001), and interactivity (β = 0.516, *p* < 0.001) all positively influenced social presence. Therefore, H1, H2, and H3 are supported. The results of the data analysis show that social presence positively influences viewing intention (β = 0.634, *p* < 0.001). Therefore, H4 is supported. The results are shown in Figure 2. In addition, a bootstrapping technique was used to examine indirect effects. The results are shown in Table 6. The results show that sense of community (β = 0.172, *p* < 0.01), emotional support (β = 0.124, *p* < 0.05), and interactivity (β = 0.224, *p* < 0.001) all have indirect effects on watching intention.

**TABLE 6:** Standardized indirect effects and 95% confidence intervals.

Path	Estimated	*P*-value	95% confidence interval	
			Lower	Upper
Sense of community → Watching intention	0.172	0.009**	0.115	0.263
Emotional support → Watching intention	0.124	0.011*	0.065	0.179
Interactivity → Watching intention	0.224	***	0.136	0.372

*p < 0.05, **p < 0.01, ***p < 0.001.

However, the variation of the standardized path coefficients between different variables is significant. Among the factors influencing viewers’ social presence, interactivity has the most significant effect on the social presence (0.516), followed by the impact of sense of community on the social presence (0.423), whereas emotional support has a lower effect on the social presence (0.384). Although the results of data analysis and literature studies (Wohn et al., 2018) suggest that emotional support enhances viewers’ social presence to some extent, the high frequency of information interaction between live streaming has weakened the effect of emotional support.

### Moderating Test

To test the moderating effect of group streamer attractiveness on the hypothetical model, we divide the collected sample data into groups with lower streamer attractiveness and higher streamer attractiveness levels. In this study, the sample data were split into groups of lower streamer attractiveness and higher streamer attractiveness according to the mean of streamer attraction (Bradburn et al., 2003). A total of 171 viewers (44.30% of the total sample) with low streamer attractiveness scored below the median of the question, whereas 215 viewers (55.70% of the total sample) with high streamer attractiveness were those who scored above the median of the question.

This study constructed a basic model without any constraints and a restricted model with equal weights of the limited test path structure. A Chi-square test was conducted to test and derive the differences in the path coefficients between different levels of streamer attractiveness. According to Shanahan et al. (2012), the moderating relationship is significant when the difference in χ^2^ (Δχ^2^) with a degree of freedom (df) of 1 is more significant than the critical value of 3.84. As shown in Table 7, the moderating effect of streamer attractiveness on the path of watching intention is substantial in the social presence (Δχ^2^ = 5.299, *p* < 0.01). And the moderating effect is positive from different grouping path coefficients. Therefore, streamer attractiveness has a positive moderating effect on the relationship between social presence and watching intention. So, hypothesis H5 is supported.

**TABLE 7:** Results of multi-group analysis.

Path	Δχ^2^	Path factor	
		Low streamer attractiveness	High streamer attractiveness
Social presence → Watching intention	5.299**	0.41***	0.52***

**p < 0.01, ***p < 0.001.

## Discussion and Implications

### Implications for Research

First, live streaming is a new research topic. Although it has been studied by scholars from different perspectives (Hu et al., 2017; Hilvert-Bruce et al., 2018; Wongkitrungrueng and Assarut, 2018; Yu et al., 2018; Hou et al., 2019; Xu et al., 2019; Park and Lin, 2020), only a few empirical studies examine live streaming watching from the perspective of social presence. Thus, we contribute to the literature explaining the intention to watch live streaming by focusing on viewer social presence in the live streaming.

Second, we reveal the formation mechanism of social presence in live streaming from the perspective of the viewer experience, which provides new theoretical support and perspective to live streaming work and managers. Most previous studies on live streaming intention under social presence theory have taken three dimensions: communication, emotion, and coexistence (Xie et al., 2019). There are also studies from both emotional and cognitive perspectives. Although they provided new insights from different perspectives, they start more from viewer motivation (Hilvert-Bruce et al., 2018; Yu et al., 2018; Xu et al., 2019; Ma, 2021) and system functionality (Sun et al., 2019; Kim et al., 2020). Examining how the viewer’s experience in live streaming affects social presence is rare. Therefore, the sense of community, emotional support, and interactivity selected in this paper are more common behavioral phenomena and experience perceptions in live streaming, better explaining how viewers develop social presence. Moreover, the comparative analysis of the three variables revealed that the influence of interactivity and sense of community is stronger, further demonstrating the formation mechanism of social presence in the live streaming context.

Third, we verify the moderating role of streamer attractiveness in social presence and watch intention, explaining the effect of streamer attractiveness on viewers’ perception. A literature review reveals that most studies only consider a single cognitive and affective mechanism (Lee and Shin, 2014; Weidlich and Bastiaens, 2019; Nadeem, 2020; Obeidat et al., 2020) not better reflect the current complex and fast-changing live streaming situation. Therefore, exploring the interaction between streamer attractiveness in social presence and watching intention. In addition, the recent research on social presence lacks the research perspective on the streamers, mainly from the aspects of web design visual and auditory senses (Sun et al., 2019; Kim et al., 2020). The present study complements these research gaps. This finding indicates that the marginal benefit of enhancing streamer attractiveness significantly affects live streaming performance, thus expanding previous studies.

### Implication for Practice

First, firms should cultivate the viewers’ sense of community to create a high-quality live community. The viewers’ sense of community can improve social presence and generate positive intention. Therefore, firms should strengthen the cultivation of the sense of community of live streaming. Specifically, firms cultivate sense of community based on different types of live streaming. For example, in the live e-commerce context, firms can develop a sense of community among viewers through product sharing. And in the live game context, game strategy sharing, group play, etc., are good means. In a word, firms should build a high-quality live streaming community through useful content production. Furthermore, excellent streamers should be cultivated to enrich community content and emotional ties strengthen the sense of community.

Second, firms should improve the skills of streamers and enhance the empathy between streamers and viewers. The two most direct and significant receptors in live streaming activities are viewers and streamers. The key to the effectiveness of live streaming depends on the skills and quality of streamers. Hence, firms need to provide regular training and skill quality training for the streamer group according to their personal attributes to develop different skills, such as communication style, humor, and physical attractiveness. Firms should focus on streamer image management to improve the attractiveness of streamers, thereby attracting faithful and loyal fans for the live streamer.

Finally, firms should strengthen the interaction and communication in the live streaming community. The rapid development of live streaming cannot be separated from people’s intrinsic motivation to reduce loneliness. Therefore, firms should strengthen the interaction between streamers and viewers. For example, streamers can get prizes if they answer streamers’ questions. Firms can also create identity tags and status symbols unique to highly interactive fans. In addition, firms need to actively care about viewers’ emotional state communicate and interact more with viewers.

### Limitations and Future Research

This article has limitations and can be extended in several ways. First, given the different types of live streaming (e.g., tourism live streaming and game live streaming) that meet viewers’ varying needs, future research can subdivide live streaming into different groups to generalize and obtain more fine-grained findings in different live streaming contexts. Second, this paper is mainly based on online questionnaire data. Third, in the future, researchers can obtain viewers’ longitudinal live streaming watching data to explore the dynamic drivers of viewers’ watching behaviors. Finally, the relationship between live streaming watching and purchase behaviors is an interesting research topic worthy of future research.

## Data Availability Statement

The original contributions presented in the study are included in the article/supplementary material, further inquiries can be directed to the corresponding author/s.

## Author Contributions

JC and JL worked together on the manuscript. JC was responsible for writing and data analysis. JL was responsible for project management and revision of the edited manuscript.

## Conflict of Interest

The authors declare that the research was conducted in the absence of any commercial or financial relationships that could be construed as a potential conflict of interest.

## Publisher’s Note

All claims expressed in this article are solely those of the authors and do not necessarily represent those of their affiliated organizations, or those of the publisher, the editors and the reviewers. Any product that may be evaluated in this article, or claim that may be made by its manufacturer, is not guaranteed or endorsed by the publisher.
